# Navigating Virtual Reality in Stroke Rehabilitation: Scoping Review of Diverse Intervention Effects

**DOI:** 10.2196/72498

**Published:** 2026-04-08

**Authors:** Ruixue Liu, Beibei Lin, Yanhuan Zhai, Wei Wang

**Affiliations:** 1 Nursing Department, The First Affiliated Hospital, Zhejiang University School of Medicine, 79 Qingchun Road, Hangzhou, China, 86 18268130750

**Keywords:** stroke, virtual reality, rehabilitation training, upper limb function, gait balance, quality of life

## Abstract

**Background:**

Virtual reality (VR) technology has been increasingly explored in stroke rehabilitation due to its immersive and interactive features. However, considerable heterogeneity exists in intervention designs, study populations, and outcome measures, limiting the feasibility of conducting a systematic review.

**Objective:**

This scoping review aims to comprehensively map randomized controlled trials (RCTs) investigating the use of VR interventions in stroke rehabilitation, with particular focus on upper limb function, gait and balance, cognitive function, and quality of life.

**Methods:**

Following the Arksey and O’Malley framework and PRISMA-ScR (Preferred Reporting Items for Systematic Reviews and Meta-Analyses extension for Scoping Reviews) guidelines, we conducted a scoping review of RCTs published in databases from their inception to January 4, 2025. Seven databases were searched, including PubMed, Embase, Web of Science, CNKI, Wanfang, VIP, and CBM. Studies were included if they met predefined eligibility criteria, including adult patients with stroke receiving VR-based rehabilitation and a randomized controlled trial design. Exclusion criteria included non-Chinese or non-English literature, literature with unavailable full text, studies with duplicate publication or data, and studies that were irrelevant to the research topic or did not incorporate VR technology in their intervention measures. Data extracted included intervention type, sample size, training frequency and duration, outcomes, and study setting. Due to significant heterogeneity across studies, a narrative synthesis approach was used. No formal risk of bias or quality appraisal was conducted.

**Results:**

Fifteen RCTs involving 804 patients with stroke were included. Intervention modalities varied significantly in terms of type, content, frequency, and duration. Nonimmersive VR (NIVR) interventions were more frequently applied in studies targeting upper limb function and gait training, while fully immersive VR (FIVR) was assessed in 2 head-mounted display (HMD)–based trials, whereas most studies used NIVR for upper-limb and gait-related outcomes. Many studies reported positive trends in motor function, cognitive performance, gait balance, and quality of life. However, findings were inconsistent, and not all outcomes reached statistical significance. Mild adverse events, such as fatigue or dizziness, were occasionally reported; however, no serious events occurred.

**Conclusions:**

This scoping review outlines the research status of VR in stroke rehabilitation. VR may offer potential benefits; however, existing studies have limitations, including substantial heterogeneity in intervention protocols, limited long-term follow-up, and baseline imbalances in some trials. In addition, because this review did not include a formal quality or risk-of-bias assessment, the observed effects should be interpreted as preliminary signals rather than definitive evidence of efficacy, and the certainty of the evidence cannot be determined. Future research should standardize outcome measures, improve methodological rigor, and incorporate quality and risk-of-bias evaluation to strengthen the evidence base and support clinical implementation.

## Introduction

Stroke is a disease with high incidence, high disability rate, and high recurrence rate [1-4], and it is one of the leading causes of death and long-term disability worldwide [5]. According to the World Health Organization (WHO), approximately 15 million people globally suffer from stroke each year [6,7], with about one-third of patients left with varying degrees of functional impairment, including limited physical activity [8,9], decreased cognitive ability, and significantly reduced quality of life. Stroke rehabilitation is an important issue in clinical medicine [10] and a critical step for patients to return to normal life [11]. Although traditional rehabilitation methods, such as physical therapy and occupational therapy, have achieved significant results in restoring basic functions, these methods have limitations in stimulating patient initiative and enhancing effectiveness [12]. Moreover, the rehabilitation needs of different patients vary significantly, and the lack of personalized treatment plans further limits the effectiveness of rehabilitation [13].

In recent years, virtual reality (VR) technology has gradually become an emerging tool in rehabilitation medicine due to its immersion, interactivity, and customizability [14]. VR technology can simulate real or virtual environments, providing patients with a safe, efficient, and highly repeatable training platform, thus enhancing patient engagement and motivation in rehabilitation [15,16]. Existing research has shown that VR has significant potential to improve upper limb function, gait balance, and cognitive abilities in patients with stroke [17]. For example, nonimmersive virtual reality (NIVR) systems can improve patients’ motor coordination and goal-directed behavior through screen display and motion-sensing interaction; fully immersive virtual reality (FIVR) systems, on the other hand, use head-mounted displays (HMDs) and motion capture technology to provide patients with highly realistic virtual environments [18], enhancing their cognitive abilities and spatial awareness. In this review, FIVR specifically refers to HMD-based systems; interventions delivered via screens or monitors without an HMD were considered NIVR. However, systematic research on the specific efficacy and applicability of different types of VR technology is still lacking. Furthermore, the cost of VR equipment, its technological complexity, and issues with patient compliance also limit its widespread adoption in clinical practice.

The application of VR in stroke rehabilitation has expanded rapidly in recent years, demonstrating promising potential in facilitating functional recovery. However, this evidence base remains fragmented, with several unresolved issues. Significant heterogeneity exists across studies in terms of intervention design, outcome measures, and patient characteristics, limiting comparability and generalizability. Most studies lack multidimensional and comprehensive evaluation frameworks, relying predominantly on single functional outcomes. Furthermore, the specific advantages of FIVR versus NIVR systems at different stages of rehabilitation remain unclear [19], with some findings even appearing contradictory [20]. Long-term effects, individualized adaptation strategies, and potential adverse events associated with VR interventions have also not been systematically explored [21]. These gaps highlight a disconnect between emerging technological applications and their clinical translation.

Given the high heterogeneity in intervention modalities, assessment tools, and study populations, a conventional systematic review remains unfeasible. This study, therefore, uses a scoping review approach to comprehensively map randomized controlled trials (RCTs) published between 2016 and 2025, examining the application of various VR interventions in stroke rehabilitation. It specifically evaluates the impact of immersive and NIVR on upper limb function, gait and balance, cognitive performance, and quality of life across different rehabilitation stages. By clarifying the comparative effectiveness and contextual suitability of different VR modalities, this review aims to address critical gaps in intervention comparison and mechanistic understanding. Scientifically, it provides a structured framework for future standardized research; clinically, it informs personalized rehabilitation strategies and supports the broader integration of VR technologies to enhance functional outcomes and long-term recovery in patients with stroke.

## Methods

### Defining the Research Questions

This scoping review was not registered on PROSPERO (International Prospective Register of Systematic Reviews), as scoping reviews are not consistently eligible for inclusion. Nonetheless, all methodological decisions were made a priori and adhered to throughout the review process. This study defines the research questions based on the PCC principle (Population, Concept, and Context) [22]. The methodology was guided by the scoping review framework proposed by Arksey and O’Malley, and the reporting adhered to the PRISMA-ScR (Preferred Reporting Items for Systematic Reviews and Meta-Analyses extension for Scoping Reviews) guidelines [22]. The study population (Population) consists of patients with stroke who receive VR technology interventions, including both patients with ischemic and hemorrhagic stroke aged ≥18 years. The research concept (Concept) focuses on VR-based interventions. VR [23] is a medium composed of interactive computer simulations that can sense the participant’s location and movement, providing feedback to one or more senses, allowing patients to interact with the virtual environment in a way that mimics real-life interactions. Based on the level of immersion, VR technology can be categorized into NIVR, semi-immersive, and FIVR types. NIVR [24] is presented in 2 dimensions and interacts through a computer display or gaming system; semi-immersive VR [25] creates 3D images using stereoscopic projection or fixed-angle displays; FIVR [26] allows users to interact in real-time through an HMD, typically combined with motion tracking, to achieve a high sense of presence in a virtual environment. Operationally, in this review, we classified an intervention as FIVR only when an HMD was explicitly used; screen-based systems (including motion-capture or force-platform games viewed on a monitor) were classified as NIVR. The research context (Context) is patients with stroke receiving VR interventions in hospital or home settings.

To systematically summarize the application and effects of VR in stroke rehabilitation, this study addresses the following questions: (1) What types of VR technologies are available at the time of this writing? (2) What are VR technology’s main contents and applications in stroke rehabilitation? (3) What are the rehabilitation effects, safety, and limitations of different types of VR interventions?

### Literature Search

The inclusion and exclusion criteria are presented in Table 1. To ensure a systematic and reproducible search process, we performed comprehensive searches across 7 databases, including PubMed, Embase, Web of Science, CNKI, VIP Chinese Science and Technology Journal Database, Wanfang Medical Journal Database, and China Biomedical Literature Database (SinoMed). The search period covered from database inception to January 4, 2025. Search strategies incorporated both controlled vocabulary (eg, MeSH [Medical Subject Headings]) and free-text terms. The final search strategies were peer-reviewed by two information specialists to ensure rigor and completeness. Detailed, reproducible search strings for each database are provided in Multimedia Appendix 1.

**Table 1.** Inclusion and exclusion criteria for randomized controlled trials of virtual reality–based rehabilitation in adult patients with stroke.

Criteria category	Inclusion criteria	Exclusion criteria
Population	Adult patients with stroke (≥18 years), diagnosed with ischemic or hemorrhagic stroke	Patients with pediatric (<18 years); patients with nonstroke neurological or musculoskeletal disorders
Intervention	Virtual reality (VR)–based rehabilitation interventions, including nonimmersive (NIVR), semi-immersive, or fully immersive (FIVR) systems	Non-VR rehabilitation methods, such as traditional physical or occupational therapy without VR integration
Comparator	Studies with or without control or comparator groups (eg, conventional rehabilitation, sham VR, or no treatment)	—^a^
Outcomes	Reported at least one rehabilitation-related outcome, including motor function, gait, balance, cognitive function, quality of life, or safety of the intervention	Studies without reported outcomes or unrelated to rehabilitation efficacy
Study design	Randomized controlled trials (RCTs)	Non-RCT designs such as case reports, cross-sectional studies, reviews, protocols, editorials
Language	Published in English or Chinese	Literature published in other languages without available translation
Publication type	Peer-reviewed full-text journal articles	Abstract-only publications, dissertations, conference papers, gray literature
Full-text availability	Full text available	Full text unavailable or inaccessible
Duplication	Original studies with unique data	Duplicate publications or overlapping datasets already included

a Not applicable.

Table 1 outlines the predefined inclusion and exclusion criteria applied in this scoping review of RCTs assessing VR-based rehabilitation interventions for adult patients with stroke. The review included studies published in English or Chinese between database inception and January 4, 2025, identified through comprehensive searches in 7 databases (PubMed, Embase, Web of Science, CNKI, VIP, Wanfang, and SinoMed). Eligible studies enrolled adult patients (aged 18 years or older) diagnosed with ischemic or hemorrhagic stroke and implemented VR-based interventions, including nonimmersive, semi-immersive, or fully immersive systems, either as standalone therapy or in combination with conventional rehabilitation. Studies were required to report at least one rehabilitation-related outcome such as motor function, gait, balance, cognitive function, quality of life, or safety. Nonrandomized designs, nonstroke populations, and studies without available full text or outcome data were excluded.

### Literature Screening and Data Extraction

Duplicate records were removed using EndNote X9 (Clarivate). Two types of duplication were addressed at different screening stages. The first involved technical duplicates identified by EndNote X9 based on identical bibliographic details (eg, title, author, and DOI). The second, labeled as “duplicate content” during full-text screening, referred to studies with substantially overlapping content despite differences in bibliographic metadata, for example, publications in different languages or multiple reports from the same research dataset. Such redundant publications were excluded following careful comparison to prevent data duplication bias. Two reviewers (RL and BL) independently screened studies based on predefined inclusion and exclusion criteria, with reasons for exclusion documented at each stage. Discrepancies were resolved through discussion or consultation with a third reviewer. The selection process was recorded using a PRISMA (Preferred Reporting Items for Systematic Reviews and Meta-Analyses)-compliant flow diagram (Checklist 1). Data extraction was performed using a standardized form with 5‐10 predefined variables, which were adjusted as needed to align with the study objectives. Extracted variables included study location, stroke type, sample size, intervention characteristics (type, content, setting, frequency, and duration), and outcome measures (motor function, gait and balance, cognitive function, health-related quality of life, and safety). Extraction was conducted independently by 2 reviewers (RL and BL) and cross-checked for consistency. As a scoping review, this study aimed to map this evidence rather than assess methodological quality or synthesize effect sizes; thus, no risk of bias or quality assessment was performed.

### Heterogeneity Handling

Due to significant heterogeneity in intervention types, equipment usage, and outcome measure assessments across studies, this study did not perform a meta-analysis but used a descriptive approach to summarize the results. The potential impact of heterogeneity on the study outcomes is further discussed in the discussion section.

## Results

### Literature Screening Process and Results

**Figure 1.**
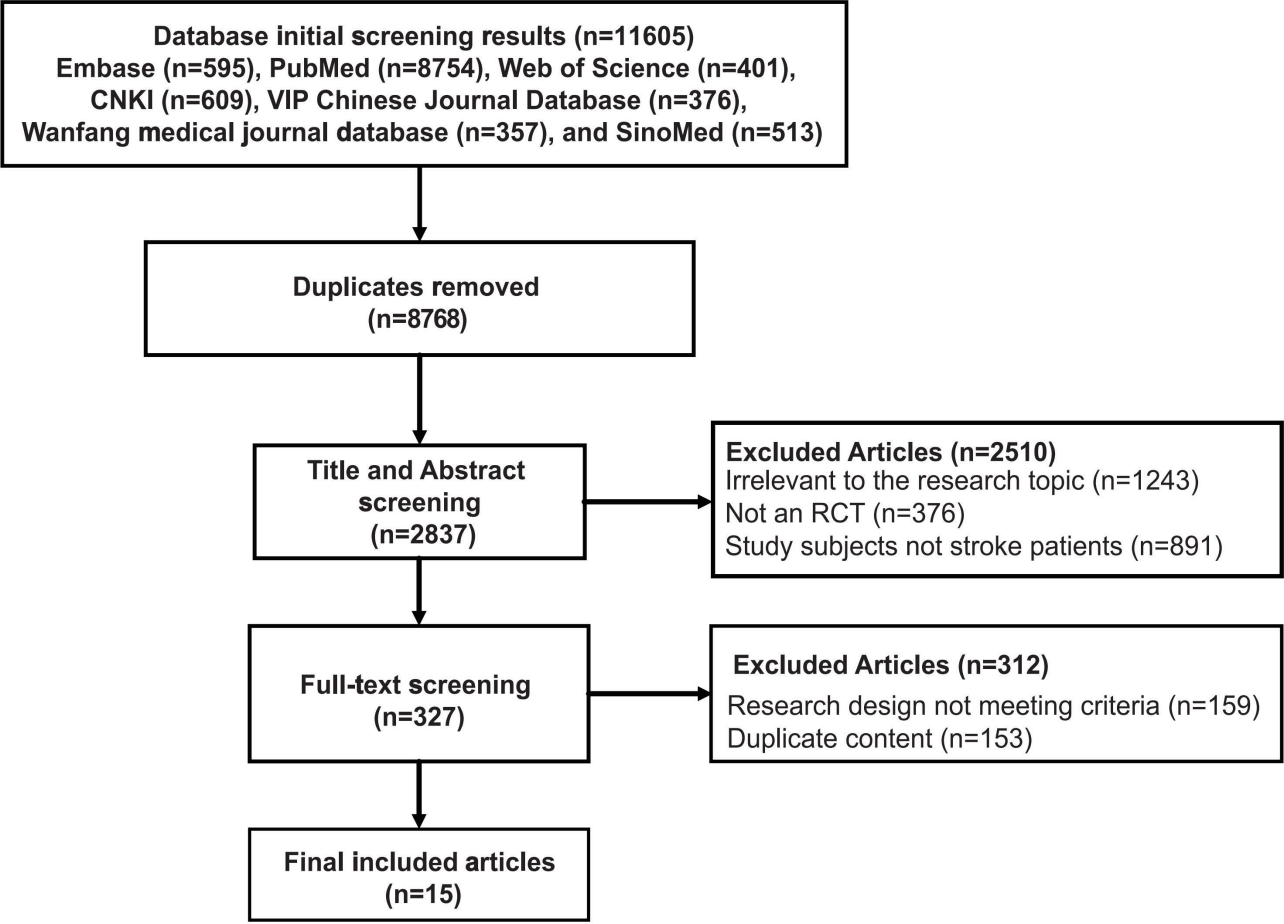
PRISMA (Preferred Reporting Items for Systematic Reviews and Meta-Analyses) flow diagram of literature screening for the scoping review of virtual reality–based rehabilitation in stroke patients. RCT: randomized controlled trial; VR: virtual reality.

A total of 11,605 records were initially identified. After removing 8768 duplicates, 2837 records remained for title and abstract screening. At this stage, 2510 records were excluded for being unrelated to the study topic, leaving 327 articles for full-text assessment. Of these, 312 were excluded, 159 due to inappropriate study design and 153 due to content duplication. Ultimately, 15 studies were included in the review (13 English articles and 2 Chinese articles) [27-41]. The specific screening process is documented in Figure 1.

### Basic Characteristics of the Included Studies

A total of 15 RCTs [27-41] were included in this study, involving 804 patients with stroke aged 45-85 years. The studies were published between 2016 and 2024. The included studies were classified and summarized based on intervention types, patient stages, and research objectives to present the study characteristics (Multimedia Appendix 2) [27-41]. Among these, 5 studies [27,32-34,41] compared VR training alone with conventional rehabilitation training; 10 studies [28-31,35-40] compared conventional rehabilitation training combined with FIVR or NIVR with conventional rehabilitation training. Nine studies [28,29,31-33,35,36,39,40] focused on upper limb rehabilitation; 4 studies [27,33,34,38] assessed cognitive or psychological outcomes. Among them, 2 studies [27,34] focused on cognitive function and anxiety improvement, 3 studies [37,38,41] focused on lower limb gait and balance improvement, and 1 study [30] focused on safety and feasibility assessment. Three studies [30,34,36] reported adverse events such as fatigue, transient dizziness, and shoulder pain, all alleviated after rest, with no severe adverse events reported (see Table 2).

**Table 2.** Forms and effects of virtual reality interventions in stroke rehabilitation research.

VR^a^ form	Core feature	Applied research number	Main application target
Non-fully immersive (screen-based/non-HMD^b^)	Use 2D screen, game controller	13	Improved upper limb function and gait balance
Full immersion (HMD-based)	Simulate a virtual environment using an HMD	2	Cognitive improvement and homeostasis improvement

a VR: virtual reality.

b HMD: head-mounted display.

Table 2 summarizes the forms and functional applications of VR interventions reported in stroke rehabilitation research. Data were synthesized from 15 randomized controlled trials conducted between 2016 and 2024 across 9 countries, including China, Spain, Canada, Turkey, and Australia. Nonimmersive VR systems, typically based on 2D screen interaction, were primarily used for upper-limb motor and gait-balance training. Fully immersive VR systems using head-mounted displays were evaluated in a limited number of trials (n=2); therefore, evidence is insufficient to draw firm conclusions regarding modality-specific application patterns (eg, cognitive or integrative rehabilitation). The evidence indicates that different levels of immersion may be associated with distinct therapeutic goals and patient suitability, and modality-specific trends should be interpreted cautiously given the small number of HMD-based FIVR trials.

Overall, the majority of included studies (n=9 [28,29,31-33,35,36,39,40]) focused on upper limb rehabilitation, suggesting the potential of VR technology to support fine motor and strength training. Several studies reported improvements in gait and balance, indicating possible benefits of VR-assisted rehabilitation for dynamic postural control. However, substantial heterogeneity remains across study designs and outcomes, underscoring the need for further high-quality trials. A small number of studies investigated cognitive and psychological outcomes, though this evidence in these domains remains limited.

### Differences in the Intervention Effects and Applicability of NIVR and FIVR

**Figure 2.**
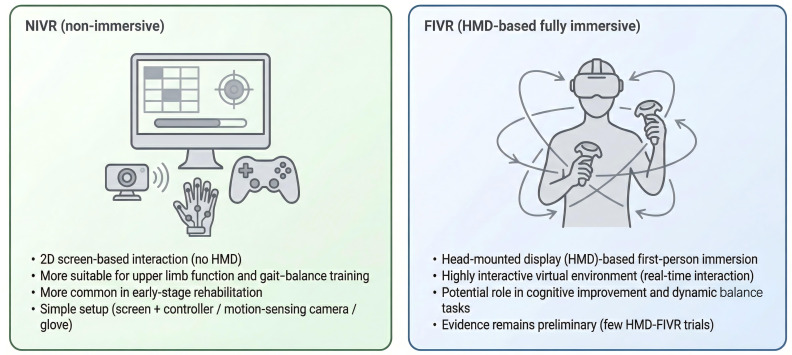
Comparison of nonimmersive and fully immersive virtual reality in stroke rehabilitation. FIVR: fully immersive virtual reality.

Among the 15 RCTs included in this study, two types of VR interventions were mainly explored: NIVR and FIVR. Each type played a different role in the rehabilitation of patients with stroke. The applications of NIVR and FIVR in rehabilitation varied, showing different effects based on the research goals and intervention stages (Figure 2).

NIVR intervention was applied in 13 studies [27-33,36-41] primarily targeting upper limb function and gait-balance training. These interventions commonly used 2D screens, customized game controllers, or training gloves. Several studies reported improvements in upper limb flexibility, strength, and gait stability, indicating potential utility across rehabilitation stages, although the timing of application varied across trials.

In contrast, FIVR intervention was applied in 2 studies [34,35], in which cognitive and/or integrative functional outcomes were assessed, often within highly interactive virtual environments. Findings indicated potential benefits in attention, visuomotor coordination, and dynamic postural control; however, given the limited number of HMD-based FIVR trials, evidence remains insufficient to draw firm conclusions regarding modality-specific application patterns or comparative advantage (Table 2).

The study subjects included acute, subacute, and patients with chronic stroke, including both ischemic and hemorrhagic stroke types. One study [41] focused on acute patients with stroke; the study is a force platform-screen interactive VR training, 10 studies [28,30,32,34-40] focused on subacute patients with stroke, and 4 studies [27,31,33,37] focused on patients with chronic stroke. Only one study [33] occurred at home, while the rest were conducted in hospitals. The included studies came from various countries, including China [28,35,39,41], Spain [29,31], Turkey [30,37], Canada [32], the United Kingdom [34], France [36], Italy [38], Norway [39], and Australia [33], ensuring a certain level of representativeness.

The application range and effects of the 2 VR forms in rehabilitation demonstrate their respective advantages and characteristics. NIVR, due to its simple equipment and ease of operation, is widely used to recover motor function and gait balance. In contrast, FIVR offers a more immersive and dynamic cognitive and balance training experience through its high level of immersion and complex virtual environments. Based on this evidence, each form of VR intervention exhibits specific strengths depending on rehabilitation goals and stages. Selection of the appropriate modality should be tailored to individual patient needs, and future studies are needed to further clarify their comparative effectiveness and clinical applicability.

### Training Platforms and Content

Three studies [30-32] used commercial games based on the Nintendo Wii (Nintendo) and Xbox Kinect (Microsoft Corp) for rehabilitation training. Games based on Nintendo Wii included card games, bingo, jigsaw puzzles, and ball games. For upper limb rehabilitation, the Wii Sports package (bowling, golf, and tennis) was used, while for lower limb rehabilitation, the Wii Fit balance training package (header ball, skiing obstacle course, tilt platform, wire tension, downstream, and ice fishing) was used. Games based on the Kinect Sports software package included bowling and “Whac-A-Mole.”

Eleven studies [27-29,33-40] used customized rehabilitation games. These included (1) functional software and hand-tutor gloves that simulate everyday tasks that patients with strokes must complete. During practice, visual and auditory feedback is provided for success or failure, scores are displayed, and therapists can adjust the sensitivity of movements to prevent frustration and reduce motivation loss. (2) The cognitive rehabilitation program (VIRTUE program) was designed after close consultation between clinical experts and patient representatives. Patients, with the help of a therapist, use VIRTUE to complete tasks in various scenarios, such as in the bedroom (making the bed and choosing clothes); the bathroom (brushing teeth and showering); the kitchen (toasting bread, preparing tea, washing dishes, cooking pasta, and using a coffee machine); the café (choosing a set meal and paying the bill); a restaurant (interacting with the waiter, ordering, or paying); and a garden (watering plants). The primary requirement is for patients to perform all or part of the activities of daily living in appropriate environments, with necessary materials such as food, kitchen utensils, and coins easily accessible while immersed in the environment. (3) Rehabilitation training using a posture control system (BioFlex-FP), including tasks like “Big Fish Eats Little Fish”, this task focuses on training weight shift in the front-back and left-right directions while also training muscle strength and endurance; “Phoenix Flying”, this task primarily targets the patient’s static balance; “Mushroom Picking”, this task mainly trains balance ability for weight shifting in all directions, stepping, and shifting weight vertically. (4) In a virtual kitchen, patients control a pan handle to cook dumplings and noodles. In a virtual fencing hall, they control a sword to burst balloons; in a virtual boxing ring, they control a large fist to hit a dummy; on a virtual basketball court, they collect eggs in a virtual basket using a controller; and in a virtual office, they organize the desk, moving items to designated positions, and perform specific shoulder joint flexion angle (30°, 60°, and 90°) training. Two studies [37,38] used 2D screen-based running and gait training, such as using the Lokomat gait orthosis to guide subjects in leg movements within the sagittal plane of the hip and knee joints, providing haptic feedback.

### Frequency and Duration of VR Interventions

In the 15 studies included, the frequency and duration of VR interventions varied depending on the intervention content (Table 3). Ten studies [27,29,30,32,34-36,38-40] required 5 VR training sessions per week, 4 studies [28,31,33,37] required 2-4 sessions per week, and one study [41] required 6 sessions per week. The training duration was 1 hour per session in 5 studies [30,32,35,36,39], 20‐45 minutes per session in 6 studies [27,33,37,38,40,41], 2.5‐3.5 hours per session in 3 studies [28,29,31], and one study [34] used personalized session durations. Additionally, the total intervention duration also varied. One study [28] had a total duration of 56 hours, one study [29] had a total duration of 30‐40 hours, 4 studies [30,31,36,38] had total durations of 20‐30 hours, 6 studies [27,32,33,35,39,40] had total durations of 10‐20 hours, 2 studies [37,41] had a total duration of 9 hours, and one study [34] had personalized intervention duration.

**Table 3.** Distribution of intervention frequency and duration in relevant rehabilitation studies.

Intervention frequency	Number of documents	Single duration	Number of documents	Total time	Number of documents
2‐4 times a week	4	20‐45 minutes/time	6	9 hours	2
5 times a week	10	1 hour/time	5	10‐20 hours	6
6 times a week	1	2.5‐3.5 hour/time	3	20‐30 hours	4
Personalized frequency	0	Personalized duration	1	30‐40 hours	1
—^a^	—	—	—	56 hours	1
—	—	—	—	Personalized total time	1

a Not applicable.

Table 3 provides a systematic summary of dosage characteristics of VR rehabilitation interventions across 15 RCTs, including training frequency, single-session duration, and total intervention time. The included studies were published between 2016 and 2024 and were conducted across 9 countries, including China, Spain, Canada, Turkey, and Australia, covering a broad range of clinical and rehabilitation settings. The VR protocols encompassed both nonimmersive and fully immersive systems and were mainly compared with conventional rehabilitation or implemented as an adjunct to standard therapy. Considerable variability was observed in intervention dosage: training frequency ranged from 2 to 6 sessions per week; single-session duration varied from 20 minutes to 3.5 hours, depending on the VR system type, training objectives, and patients’ functional status; and total intervention time ranged from 9 to 56 hours, with one study reporting a personalized total intervention duration. Overall, this table offers an independent and comprehensive overview of VR dosage designs in contemporary poststroke RCTs and highlights substantial differences in implementation strategies, training intensity, and clinical applicability across settings and patient populations.

The results suggest that the VR intervention frequency and duration design should be adjusted based on the patient’s rehabilitation stage and specific goals. A common and effective approach is to conduct 3-5 weekly sessions, each lasting 20 minutes to 1 hour in duration. High-frequency training is suitable for patients in the acute phase, while personalized plans offer potential solutions for more complex cases.

### Effects of VR Interventions in Stroke Rehabilitation and Heterogeneity Analysis

This study evaluated 15 studies that assessed the effects of VR interventions on various aspects of stroke rehabilitation, including motor function, health-related quality of life, gait and balance, cognitive and psychological status, and safety and feasibility. Notably, although every included study reported at least one statistically significant finding, several outcome measures within individual studies did not reach statistical significance.

Regarding motor function, 9 studies [27-32,34-36] assessed improvements in upper and lower limb function. In these studies, screen-based NIVR was frequently used to support motor training through interactive feedback. Because only a small number of trials used HMD-based FIVR, this evidence is insufficient to conclude modality-specific preferences (eg, upper- vs lower-limb targets) or comparative advantages.

Regarding health-related quality of life, 9 studies [27,28,31,32,34-36,38,39] investigated the effects of VR interventions on patients’ quality of life, with the primary measurement tools being the short form health survey 36 (SF-36) and stroke impact scale (SIS). Some studies found that VR interventions, whether applied alone or in combination with conventional rehabilitation, may be associated with improvements in patients’ quality of life, particularly showing positive trends in daily functional independence and mental well-being.

The improvement of gait and balance was the focus of 3 studies [31,37,38], using tools such as the Berg Balance Scale and Functional Gait Assessment (FGA). These predominantly screen-based interventions showed mixed but potentially beneficial effects on postural control. However, substantial heterogeneity exists across studies. Regarding cognitive and psychological status, 2 studies [27,34] explored the effects of VR interventions on cognitive function and anxiety relief, using the Montreal Cognitive Assessment (MoCA) and the Hospital Anxiety and Depression Scale (HADS) as measurement tools. Some studies suggested that VR interventions may be associated with changes in cognitive attention and psychological status, particularly during complex task training delivered in interactive virtual settings. While certain findings indicate positive trends, the overall evidence remains limited, and the specific contribution of HMD-based immersion cannot be determined given the small number of HMD-based FIVR trials.

Regarding safety and feasibility, 3 studies [30,34,36] reported mild adverse events, including fatigue, transient dizziness, and shoulder pain, all of which were alleviated with rest, and no serious adverse events occurred. The included studies indicate that VR interventions in patients with stroke appear to be generally safe and feasible; however, the limited sample sizes and insufficient reporting details warrant further investigation to confirm these findings. However, there was significant heterogeneity in the interventions, outcome measurement tools, and patient characteristics. Specifically, the target functions of NIVR and FIVR differ, and the use of measurement tools was inconsistent across studies. This heterogeneity may have contributed to inconsistencies in the outcome measures. Future research should address this issue by standardizing intervention protocols and unifying measurement tools.

## Discussion

### Principal Findings

In recent years, VR technology has emerged as an innovative approach to rehabilitation, gaining widespread attention in the medical field [16]. Previous studies have reported that VR can provide an immersive and highly interactive training environment for stroke rehabilitation [42], showing potential value in improving motor function, enhancing cognitive abilities, and increasing quality-of-life-related outcomes [42-45]. Following a stroke, patients often experience limb dysfunction, gait and balance impairments, and cognitive deficits. Traditional rehabilitation methods frequently lack sufficient diversity and personalized intervention.

This review highlights that as a novel adjunct therapy, VR’s unique virtual interaction and real-time feedback mechanisms may play a crucial role in addressing these challenges. However, although numerous studies have reported positive rehabilitation outcomes with VR intervention, the effectiveness of different types of VR interventions and their specific applications remains insufficiently validated. This represents a key challenge in the field of stroke rehabilitation. Existing studies have shown that NIVR and FIVR interventions have distinct effects and scopes of application in stroke rehabilitation. NIVR, characterized by its simplicity and ease of use, is more commonly applied in the early stages of rehabilitation, particularly in upper limb recovery and gait-balance training. This aligns with its use of basic interactive and feedback mechanisms, often delivered through gamified tasks to enhance patient motivation and engagement. In contrast, HMD-based FIVR has been investigated in only a small number of trials (n=2) to explore potential effects on cognitive function and dynamic balance. Although highly interactive virtual environments may enhance immersion and user engagement, the evidence remains preliminary, and it is not possible to draw firm conclusions regarding modality-specific advantages or preferential application domains (eg, lower limb function or cognitive attention).

### Comparison to Prior Work

The studies included in this review demonstrate substantial heterogeneity in this VR intervention research. Variations in intervention protocols, assessment tools, and individual patient characteristics contribute to inconsistencies in outcome measures. For example, the target populations and training objectives differ between NIVR and FIVR applications. The selection of evaluation instruments, such as functional gait assessments and cognitive measurement tools, is not standardized across studies, which may account for differences in reported outcomes. Additionally, variability in intervention frequency and duration may impact overall effectiveness. Most studies adopted protocols involving three to five sessions per week, with each session lasting 20 minutes to 1 hour. Although some studies have reported positive effects on multiple rehabilitation outcomes, particularly in patients with acute-phase conditions and those with complex conditions, research on personalized intervention strategies remains limited and should be prioritized in future investigations.

VR technology represents a novel adjunct in stroke rehabilitation. Existing studies have explored its use in promoting motor function and neural engagement. NIVR interventions have predominantly focused on upper limb function and gait and balance, while FIVR has been associated with cognitive and dynamic postural training. These effects may also contribute to increased training adherence and improvements in quality of life. Most studies did not report serious adverse events, and VR interventions were generally considered safe. Mild adverse effects, such as fatigue, transient dizziness, and shoulder discomfort, were occasionally reported. However, the high cost and technical complexity of FIVR systems may limit their clinical use, especially in resource-limited settings. Reducing equipment costs and improving usability are key areas for future development. The considerable heterogeneity among the included studies, in terms of VR intervention types, assessment tools, and patient profiles, may have affected the consistency of the findings.

### Limitations

While VR interventions have shown promise in stroke rehabilitation, several limitations remain. First, significant heterogeneity exists across studies in terms of device types, training content, frequency, and duration, which may impact the consistency of outcomes. Second, most included RCTs were short-term and lacked follow-up, limiting understanding of long-term efficacy. Third, some studies did not match baseline characteristics well, potentially underestimating individual variability. Fourth, older adults and individuals with cognitive impairments may experience difficulty interacting with complex VR systems, which could compromise engagement and the intervention’s effectiveness. Developing simplified and adaptive interfaces could help mitigate this issue and improve accessibility. Fifth, this review did not conduct a formal assessment of methodological quality or risk of bias for the included RCTs (eg, using the Cochrane RoB tool or the Physiotherapy Evidence Database [PEDro] scale). Therefore, the synthesized effects and between-group differences should be interpreted as preliminary and indicative, reflecting patterns and trends in the evidence rather than definitive efficacy conclusions. Future systematic reviews should incorporate standardized quality and risk-of-bias appraisal to strengthen the reliability and robustness of the evidence.

### Future Directions

Future research should further explore the differential effects of VR interventions across various stroke subgroups, particularly between acute and chronic phases. Given the heterogeneity in recovery trajectories, developing personalized intervention strategies is essential. Selecting appropriate types of VR, such as nonimmersive versus fully immersive systems, based on specific patient needs could enhance clinical relevance and therapeutic outcomes. Additionally, integrating VR with conventional rehabilitation modalities, such as physical therapy, electrical stimulation, and robotic training, may offer synergistic benefits.

To improve comparability and rigor across studies, the adoption of standardized assessment tools and outcome measures is recommended. Moreover, the potential of emerging technologies such as brain-computer interfaces, artificial intelligence, and telerehabilitation platforms warrants further exploration, particularly for enhancing the precision and individualization of interventions. Finally, to increase accessibility, especially in resource-limited or home-based settings, future efforts should focus on reducing equipment costs, simplifying user interfaces, and improving system usability.

### Conclusion

**Figure 3.**
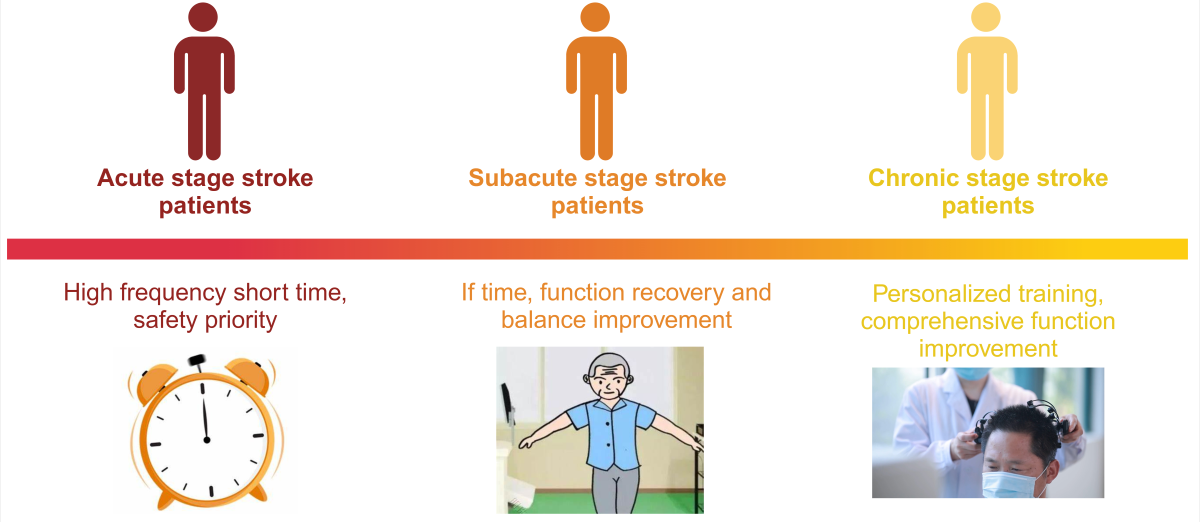
Virtual reality intervention strategies for different stroke recovery stages.

This scoping review highlights the growing role of VR as an innovative adjunct to conventional stroke rehabilitation. This evidence indicates that VR-based interventions may be associated with improvements in upper limb function, gait and balance, cognitive performance, and health-related quality of life, with a generally favorable safety profile (Figure 3). Across the included trials, NIVR was more commonly applied to upper limb rehabilitation and gait-balance training, particularly in earlier rehabilitation stages. HMD-based FIVR was examined in only a small number of trials; therefore, any modality-specific observations regarding cognitive rehabilitation or dynamic postural tasks should be considered preliminary and interpreted with caution. These modality-specific observations should be interpreted as potential trends rather than confirmed comparative superiority, given the substantial heterogeneity in intervention protocols, outcome measures, and patient characteristics, as well as the limited evidence on long-term outcomes. A comparative overview of VR intervention types, functional targets, and application characteristics is presented in Table 4.

**Table 4.** Summary of key findings and insights in virtual reality–based stroke rehabilitation research.

Content category	Main findings and insights
Research background	VR^a^ technology has attracted increasing attention in stroke rehabilitation due to its strong immersion and high interactivity.
Differences in intervention types	Nonimmersive VR is more suitable for upper limb function and gait-balance training, whereas HMD^b^-based fully immersive VR has been investigated in only a small number of trials; its potential role in cognitive improvement and dynamic balance tasks remains preliminary.
Effectiveness evaluation	Most studies suggest that VR interventions improve motor function, cognitive ability, and quality of life, with generally high safety and mild side effects.
Heterogeneity in Research	Significant inconsistencies exist in VR intervention protocols, assessment tools, and individual characteristics, limiting the comparability of results.
Limitations	Lack of long-term follow-up, standardized assessment tools, and high-quality RCTs^c^; some populations show poor adaptability to VR.
Future Directions	Future research should focus on personalized intervention strategies, integration with AI^d^ and BCI^e^ technologies, and enhancing accessibility and home-based applications.

a VR: virtual reality.

b HMD: head-mounted display.

c RCT: randomized controlled trial.

d AI: artificial intelligence.

e BC: brain-computer Interface.

## Supplementary material

10.2196/72498 Multimedia Appendix 1 Search strategies.

10.2196/72498 Multimedia Appendix 2 Characteristics of the included studies (n=15).

10.2196/72498 Checklist 1 PRISMA checklist.

## Abbreviations

FGA: Functional Gait Assessment
FIVR: fully immersive virtual reality
HADS: Hospital Anxiety and Depression Scale
HMD: head-mounted display
MeSH: Medical Subject Headings
MoCA: Montreal Cognitive Assessment
NIVR: nonimmersive virtual reality
PCC: Population, Concept, and Context
PRISMA: Preferred Reporting Items for Systematic Reviews and Meta-Analyses
PRISMA-ScR: Preferred Reporting Items for Systematic Reviews and Meta-Analyses Extension for Scoping Reviews
PROSPERO: International Prospective Register of Systematic Reviews
RCT: randomized controlled trial
SF-36: Short Form Health Survey-36 item
SIS: Stroke Impact Scale
VR: virtual reality
WHO: World Health Organization
