# Supplementary material for: Navigating Virtual Reality in Stroke Rehabilitation: Scoping Review of Diverse Intervention Effects
Source: JMIR Serious Games. 2026 Apr 8;14:e72498. doi: 10.2196/72498 (PMC13061288; doi:10.2196/72498)
Supplement: Multimedia Appendix 1 [file games-v14-e72498-s001.docx]

# Multimedia Appendix 1: Search Strategies.

| **Database** | **Coverage Period** | **Search Date** | **Search Strategy** |
| --- | --- | --- | --- |
| PubMed | Inception to Jan 4, 2025 | Jan 4, 2025 | ("Stroke"[MeSH] OR "Stroke"[Title/Abstract] OR "cerebrovascular accident"[Title/Abstract] OR "CVA"[Title/Abstract]) AND ( "Virtual Reality"[MeSH] OR "virtual reality"[Title/Abstract] OR "VR"[Title/Abstract] OR "virtual reality therapy"[Title/Abstract] OR "Augmented Reality"[MeSH] OR "augmented reality"[Title/Abstract] OR "AR"[Title/Abstract] OR "mixed reality"[Title/Abstract] OR "MR"[Title/Abstract] ) |
| Embase | Inception to Jan 4, 2025 | Jan 4, 2025 | ('stroke'/exp OR 'cerebrovascular accident'/exp OR stroke:ti,ab OR 'cerebrovascular accident':ti,ab OR 'CVA':ti,ab) AND ('virtual reality'/exp OR 'virtual reality':ti,ab OR 'virtual environment':ti,ab OR 'virtual simulation':ti,ab OR 'VR':ti,ab OR 'virtual reality therapy':ti,ab OR 'Exergaming':ti,ab) |
| Web of Science | Inception to Jan 4, 2025 | Jan 4, 2025 | TS=("stroke" OR "cerebrovascular accident" OR "CVA" OR "cerebral infarction" OR "cerebral hemorrhage") AND TS=("virtual reality" OR "VR" OR "augmented reality" OR "mixed reality" OR "extended reality")AND TS=("randomized controlled trial" OR "RCT" OR "clinical trial" OR "randomised controlled trial") |
| CNKI | Inception to Jan 4, 2025 | Jan 4, 2025 | Subject = (stroke OR cerebrovascular accident OR cerebral infarction OR cerebral hemorrhage OR cerebral embolism) AND Subject = (virtual reality OR virtual environment OR virtual scenario)  (Subject = (stroke OR cerebral stroke OR cerebrovascular accident OR cerebral infarction OR cerebral hemorrhage)) AND (Subject = (virtual reality OR VR OR augmented reality OR mixed reality OR extended reality)) AND (Title = randomized OR Keywords = randomized OR Abstract = randomized) |
|  |  |  | SU=(‘脑卒中’ OR ‘中风’ OR ‘卒中’ OR ‘脑血管意外’ OR ‘脑梗塞’ OR ‘脑梗死’ OR ‘脑出血’) AND SU=(‘虚拟现实’ OR ‘VR’ OR ‘增强现实’ OR ‘混合现实’ OR ‘扩展现实’) AND (TKA=(‘随机’ OR ‘随机对照’ OR ‘RCT’)) |
| VIP | Inception to Jan 4, 2025 | Jan 4, 2025 | Subject = (stroke OR cerebrovascular disease OR cerebral infarction OR cerebral hemorrhage) AND Subject = (virtual reality OR virtual environment OR VR OR virtual training) |
|  |  |  | R=(‘脑卒中’ OR ‘中风’ OR ‘卒中’ OR ‘脑血管意外’ OR ‘脑梗塞’ OR ‘脑梗死’ OR ‘脑出血’) AND R=(‘虚拟现实’ OR ‘VR’ OR ‘增强现实’ OR ‘混合现实’ OR ‘虚拟训练’) AND R=(‘康复’ OR ‘训练’ OR ‘治疗’ OR ‘干预’) |
| Wanfang | Inception to Jan 4, 2025 | Jan 4, 2025 | Subject = (stroke OR cerebrovascular accident OR cerebral infarction OR cerebral hemorrhage)  AND Subject = (virtual reality OR VR OR augmented reality OR mixed reality OR extended reality)AND (Title = randomized OR Keywords = randomized OR Abstract = randomized) |
|  |  |  | 主题:(脑卒中 OR 中风 OR 卒中 OR 脑血管意外 OR 脑梗塞 OR 脑梗死 OR 脑出血) AND 主题:(虚拟现实 OR VR OR 增强现实 OR 混合现实 OR 扩展现实) AND 主题:(随机 OR 随机对照 OR RCT) |
| SinoMed | Inception to Jan 4, 2025 | Jan 4, 2025 | (stroke OR cerebral infarction OR cerebral hemorrhage OR cerebrovascular accident OR cerebrovascular disease) AND (virtual reality OR virtual environment OR VR OR virtual rehabilitation training) |
|  |  |  | 脑卒中 OR 中风 OR 卒中 OR 脑血管意外 OR 脑梗塞 OR 脑梗死 OR 脑出血) AND (虚拟现实 OR VR OR 增强现实 OR 混合现实 OR 虚拟康复训练) AND (康复 OR 训练 OR 治疗 OR 干预) |
