# Supplementary material for: Navigating Virtual Reality in Stroke Rehabilitation: Scoping Review of Diverse Intervention Effects
Source: JMIR Serious Games. 2026 Apr 8;14:e72498. doi: 10.2196/72498 (PMC13061288; doi:10.2196/72498)
Supplement: Multimedia Appendix 2 [file games-v14-e72498-s002.docx]

**Multimedia Appendix 2: Characteristics of the included studies (n=15).**

| **Included literature** | **Country/Region** |  | **Sample Characteristics** | | **Intervention measures** | | | | | | | | **Control group intervention measures** | **Outcome indicators** | **Effect Direction (Positive/Negative)** | **Any statistically significant outcome reported (Yes/No)** |
| --- | --- | --- | --- | --- | --- | --- | --- | --- | --- | --- | --- | --- | --- | --- | --- | --- |
|  |  | Type of stroke | sample size  (Example, experimental group/control group) | Duration of onset | Training type | Training content | place | frequency | course of treatment | | |  | |  |  |  |
| Lam et al 2022 [28] | Hong Kong, China |  | 47/46 | Within 6 months | Non-immersive VR training+conventional rehabilitation therapy | Play different computer games with a customized game controller using paralyzed hands | hospital | 3.5 hours/time, 2 times/week | | 16 times | Guided exercise+routine rehabilitation | | | A① A③ A② B① | Positive | Yes |
| Rodriguez-Hernandez et al 2023 [29] | Spain | Hemorrhagic/ischemic stroke | 23/23 | Within 6 months | Non-immersive VR training+conventional rehabilitation therapy | Based on SVR device application, Handtutor gloves are used to simulate the actions and tasks that stroke survivors must complete in their daily lives | hospital | 2.5 hours/time, 5 times/week | | 15 times | Conventional rehabilitation therapy | | | A① A② A④ | Positive | Yes |
| Turkbe et al  2017 [30] | Turkey |  | 10/10 | Within 9 months | Non-immersive VR training+regular rehabilitation | Non-immersive VR (Xbox Kinect-based training) | hospital | 1 hour/time, 5 times/week | | 20 times | Conventional rehabilitation therapy | | | E A⑤  A⑦ A⑧ | Positive | Yes |
| Marques-Sule et al  2021 [31] | Spain | Ischemic Stroke | 15/14 | More than 12 months | Non-immersive VR training+conventional rehabilitation therapy | Based on Nintendo Wii, use Wii Sports game pack for upper limbs and Wii Fit balance training pack for lower limbs | hospital | 2.5 hours/time, 2 times/week | | 8 times | Conventional rehabilitation therapy | | | C⑦ A① B③ B⑤ | Positive | Yes |
| Saposni et al 2016 [32] | Canada, Argentina, Peru, Thailand |  | 71/70 | Within 3 months | Non-immersive VR training | Playing card games, bingo games, stacked music, or ball games based on the Wii Nintendo gaming system | hospital | 1 hour/time, 5 times/week | | 10 times | Conventional rehabilitation therapy | | | A⑥ A⑤ B④ A⑦  B③ A② | Positive | Yes |
| Huang et al 2023 [35] | Chinese Mainland |  | 20/20 | Within 1 month | Fully immersive VR training+regular rehabilitation | Virtual kitchen, virtual boxing training, virtual office, etc. based on VR | hospital | 1 hour/time, 5 times/week | | 15 times | Conventional rehabilitation therapy | | | A① B③ | Positive | Yes |
| Chatterjee et al 2022 [34] | Britain |  | 30/10 | Within 3 weeks | Fully immersive VR training | Pick up an object with a handheld controller based on VIRTUE and move it to a new location | hospital | Personalized intervention, 5 times per week | | 10 times | Conventional rehabilitation therapy | | | D① E  D② D④ B② | Positive | Yes |
| Laffont et al 2020 [36] | France | Hemorrhagic/ischemic stroke | 25/26 | Within 3 months | Non-immersive VR training+conventional rehabilitation therapy | Non immersive video game rehabilitation training | hospital | 1 hour/time, 5 times/week | | 30 times | Conventional rehabilitation therapy | | | A① A⑤ A⑥ B③ B①  E | Positive | Yes |
| Calabro et al 2017 [38] | Italy |  | 12/12 | Within 12 months | Non-immersive VR+robot assisted gait training (RAGT) | Running Sports Based on nIVR | hospital | 45 minutes/time, 5 times/week | | 40 times | Robot Assisted Gait Training (RAGT) | | | C① D⑤ C② | Mixed | Yes |
| Liang et al 2020 [41] | Chinese Mainland |  | 27/27 | Within 2 weeks | Non-immersive VR training (screen-based) | Rehabilitation game training based on BioFlex-FP with visual feedback on a display/monitor | hospital | 30 minutes/time, 6 times/week | | 18 times | Conventional rehabilitation therapy | | | C⑦ C⑧ | Positive | Yes |
| Brunner I et al.2017 [39] | Norway |  | 62/58 | Within 3 months | Non-immersive VR training+conventional rehabilitation therapy | Upper limb reinforcement training based on YouGrabber system | hospital | 1 hour/time, 5 times/week | | 16~20 times | Conventional rehabilitation therapy | | | A③ A⑤ A⑦ | Positive | Yes |
| Maier et al 2020 [27] | Barcelona |  | 19/19 | More than 6 months | Non-immersive VR training (screen-based) | Gamified training of multimodal tasks based on RGS delivered via screen/monitor interaction | hospital | 30 minutes/time, 5 times/week | | 30 times | Conventional rehabilitation therapy | | | D⑦ D① B③ A① D⑤ D③ | Positive | Yes |
| Li et al 2023 [40] | Chinese Mainland |  | 30/30 | Within 2 months | Non-immersive VR training+conventional rehabilitation therapy | Specific flexion angle training of shoulder joint based on BioMaster | hospital | 20 minutes/time, 2 times/day, 5 times/week | | 30 times | Conventional rehabilitation therapy | | | A① A⑨ | Positive | Yes |
| Wilson et al 2021 [33] | Australia |  | 10/8 | Within 12 months | Non-immersive VR training | Home virtual rehabilitation training based on Elements (EDNA-22) system | At home | 30 minutes/time, 3-4 times/week | | At least 24 times | GRASP Rehabilitation Training | | | A⑤ D① B④ D⑥ | Positive | Yes |
| Kayabinar et al 2021 [37] | Türkiye |  | 15/15 | More than 6 months | Non-immersive VR training+RAGT therapy | VR games based on two-dimensional screens | hospital | 45 minutes/time, 2 times/week | | 12 times | RAGT treatment | | | C① C⑦ C⑥ C⑤ | Mixed | Yes |

Note: A Physical function assessment① Fugl-Meyer Assessment of Upper Extremity(FMA-UE) ② Grip Strength(GS) ③ The Action Research Arm Test(ARAT) ④ Ashworth Scale ⑤ Box and Block Test(BBT) ⑥ Wolf Motor Function Test(WMFT) ⑦ Functional Independence Measure(FIM)⑧ Brunnstrom Motor Recovery Stage(BMRS) ⑨ Modified Ashworth scale(MAS)

B Health related quality of life assessment: ① Short Form 36(SF-36)② Quality of Life(EuroQoL)③ Barthel Index(BI)④ Stroke Impact Scale(SIS)⑤ Frenchay activity index(FAI)

C Gait balance assessment: ① Rivermead Activity Index, RMI ② Tinetti Performance Oriented Mobility Assessment (POMA) ③ Burke Lateropulsion Scale (BLS) ④ Sheikh Trunk Control Test ⑤Functional Gait Assessment (FGA) ⑥ Fall Efficacy Scale International (FES-I) ⑦ Berg Balance Scale (BBS) ⑧ 3D gait analysis);

D Assessment of Mental Cognitive Status: ①Montreal Cognitive Assessment(MoCA) ② Nottingham Extended ADL (NEADL) ③Mini-Mental State Examination (MMSE) ④ Hospital Anxiety and Depression Scale (HADS) ⑤ Hamilton Depression Rating Scale (HRS) ⑥Neurobehavioural function inventory (NFI) ⑦ Cognitive Function;

E Safety and feasibility

F: The trial by Chatterjee K et al.2022 [34] used an unequal randomization scheme with a 3:1 intervention-to-control allocation (VR n=30; sham VR n=10), as specified in the original study design. This approach increased participants’ exposure to the VR intervention to support feasibility/safety and acceptability assessment, while enabling exploratory evaluation of potential treatment effects.

References:

27. Maier M, Ballester BR, Leiva Bañuelos N, Duarte Oller E, Verschure P. Adaptive conjunctive cognitive training (ACCT) in virtual reality for chronic stroke patients: a randomized controlled pilot trial. J Neuroeng Rehabil. Mar 6, 2020;17(1):42. [doi: 10.1186/s12984-020-0652-3] [Medline: 32143674]

28. Lam SSL, Liu TW, Ng SSM, Lai CWK, Woo J. Bilateral movement-based computer games improve sensorimotor functions in subacute stroke survivors. J Rehabil Med. Jul 25, 2022;54:jrm00307. [doi: 10.2340/jrm.v54.913] [Medline: 35730901]

29. Rodríguez-Hernández M, Polonio-López B, Corregidor-Sánchez AI, Martín-Conty JL, Mohedano-Moriano A, Criado-Álvarez JJ. Can specific virtual reality combined with conventional rehabilitation improve poststroke hand motor function? A randomized clinical trial. J Neuroeng Rehabil. Apr 4, 2023;20(1):38. [doi: 10.1186/s12984-023-01170-3] [Medline: 37016408]

30. Türkbey TA, Kutlay S, Gök H. Clinical feasibility of Xbox KinectTM training for stroke rehabilitation: a single-blind randomized controlled pilot study. J Rehabil Med. Jan 19, 2017;49(1):22-29. [doi: 10.2340/16501977-2183] [Medline: 27973678]

31. Marques-Sule E, Arnal-Gómez A, Buitrago-Jiménez G, Suso-Martí L, Cuenca-Martínez F, Espí-López GV. Effectiveness of nintendo wii and physical therapy in functionality, balance, and daily activities in chronic stroke patients. J Am Med Dir Assoc. May 2021;22(5):1073-1080. [doi: 10.1016/j.jamda.2021.01.076] [Medline: 33639116]

32. Saposnik G, Cohen LG, Mamdani M, et al. Efficacy and safety of non-immersive virtual reality exercising in stroke rehabilitation (EVREST): a randomised, multicentre, single-blind, controlled trial. Lancet Neurol. Sep 2016;15(10):1019-1027. [doi: 10.1016/S1474-4422(16)30121-1] [Medline: 27365261]

33. Wilson PH, Rogers JM, Vogel K, Steenbergen B, McGuckian TB, Duckworth J. Home-based (virtual) rehabilitation improves motor and cognitive function for stroke patients: a randomized controlled trial of the elements (EDNA-22) system. J Neuroeng Rehabil. Nov 25, 2021;18(1):165. [doi: 10.1186/s12984-021-00956-7] [Medline: 34823545]

34. Chatterjee K, Buchanan A, Cottrell K, Hughes S, Day TW, John NW. Immersive virtual reality for the cognitive rehabilitation of stroke survivors. IEEE Trans Neural Syst Rehabil Eng. 2022;30:719-728. [doi: 10.1109/TNSRE.2022.3158731] [Medline: 35271448]

35. Huang Q, Jiang X, Jin Y, et al. Immersive virtual reality-based rehabilitation for subacute stroke: a randomized controlled trial. J Neurol. Mar 2024;271(3):1256-1266. [doi: 10.1007/s00415-023-12060-y] [Medline: 37947856]

36. Laffont I, Froger J, Jourdan C, et al. Rehabilitation of the upper arm early after stroke: video games versus conventional rehabilitation. A randomized controlled trial. Ann Phys Rehabil Med. May 2020;63(3):173-180. [doi: 10.1016/j.rehab.2019.10.009] [Medline: 31830535]

37. Kayabinar B, Alemdaroğlu-Gürbüz İ, Yilmaz Ö. The effects of virtual reality augmented robot-assisted gait training on dual-task performance and functional measures in chronic stroke: a randomized controlled single-blind trial. Eur J Phys Rehabil Med. Apr 2021;57(2):227-237. [doi: 10.23736/S1973-9087.21.06441-8] [Medline: 33541040]

38. Calabrò RS, Naro A, Russo M, et al. The role of virtual reality in improving motor performance as revealed by EEG: a randomized clinical trial. J Neuroeng Rehabil. Jun 7, 2017;14(1):53. [doi: 10.1186/s12984-017-0268-4] [Medline: 28592282]

39. Brunner I, Skouen JS, Hofstad H, et al. Virtual reality training for upper extremity in subacute stroke (VIRTUES): a multicenter RCT. Neurology (ECronicon). Dec 12, 2017;89(24):2413-2421. [doi: 10.1212/WNL.0000000000004744] [Medline: 29142090]

40. Li H, Lao F, Gu X. Effect of virtual reality-based shoulder flexion training at specific angles on upper limb function recovery in stroke patients with hemiplegia. Chin J Phys Med Rehabil. 2023;45(2):134-136. [doi: 10.3760/cma.j.issn.0254-1424.2023.02.009]

41. Liang S, Wu Y, Zhang R. Effect of virtual reality training on balance and gait in patients with cerebellar infarction and balance disorder. Chin J Rehabil Med. 2020;35(6):700-704. [doi: 10.3969/j.issn.1001-1242.2020.06.010]
