# Supplementary material for: Navigating Virtual Reality in Stroke Rehabilitation: Scoping Review of Diverse Intervention Effects
Source: JMIR Serious Games. 2026 Apr 8;14:e72498. doi: 10.2196/72498 (PMC13061288; doi:10.2196/72498)
Supplement: Checklist 1 [file games-v14-e72498-s003.pdf]

PRISMA 2020 Checklist Report

Generated: 2025/9/9

Completed Items: 43 of 27

Section	Item #	PRISMA 2020 Item	Reported on page #	Status
Title and abstract	1	Title	1	Complete
Title and abstract	2	Abstract	2-3	Complete
Introduction	3	Rationale	4-5	Complete
Introduction	4	Objectives	4-5	Complete
Methods	5	Eligibility criteria	6-7	Complete
Methods	6	Information sources	6-9	Complete
Methods	7	Search strategy	6-9	Complete
Methods	8	Selection process	6-9	Complete

Section	Item #	PRISMA 2020 Item	Reported on page #	Status
Methods	9	Data collection process	6-9	Complete
Methods	10a	Data items	6-9	Complete
Methods	10b	Data items	6-9	Complete
Methods	11	Study risk of bias assessment	6-9	Complete
Methods	12	Effect measures	6-9	Complete
Methods	13a	Synthesis methods	6-9	Complete
Methods	13b	Synthesis methods	6-9	Complete
Methods	13c	Synthesis methods	6-9	Complete
Methods	13d	Synthesis methods	6-9	Complete
Methods	13e	Synthesis methods	6-9	Complete

Section	Item #	PRISMA 2020 Item	Reported on page #	Status
Methods	13f	Synthesis methods	6-9	Complete
Methods	14	Reporting bias assessment	6-9	Complete
Methods	15	Certainty assessment	6-9	Complete
Results	16a	Study selection	10-18	Complete
Results	16b	Study selection	10-18	Complete
Results	17	Study characteristics	10-18	Complete
Results	18	Risk of bias in studies	10-18	Complete
Results	19	Results of individual studies	10-18	Complete
Results	20a	Results of syntheses	10-18	Complete
Results	20b	Results of syntheses	10-18	Complete

Section	Item #	PRISMA 2020 Item	Reported on page #	Status
Results	20c	Results of syntheses	10-18	Complete
Results	20d	Results of syntheses	10-18	Complete
Results	21	Reporting biases	10-18	Complete
Results	22	Certainty of evidence	10-18	Complete
Discussion	23a	Discussion	19-23	Complete
Discussion	23b	Discussion	19-23	Complete
Discussion	23c	Discussion	19-23	Complete
Discussion	23d	Discussion	19-23	Complete
Other information	24a	Registration and protocol	25	Complete
Other information	24b	Registration and protocol	25	Complete

Section	Item #	PRISMA 2020 Item	Reported on page #	Status
Other information	24c	Registration and protocol	25	Complete
Other information	25	Support	25	Complete
Other information	26	Competing interests	25	Complete
Other information	27	Availability of data, code and other materials	25	Complete

Generated by PRISMA 2020 Interactive Checklist Tool | © 2025 Editverse.com

For complete PRISMA guidelines, visit: www.prisma-statement.org
